# Solution-based targeted genomic enrichment for precious DNA samples

**DOI:** 10.1186/1472-6750-12-20

**Published:** 2012-05-04

**Authors:** Aiden Eliot Shearer, Michael S Hildebrand, Richard JH Smith

**Affiliations:** 1 Department of Otolaryngology—Head & Neck Surgery, University of Iowa Carver College of Medicine, Iowa City, IA, 52242, USA; 2 Department of Molecular Physiology & Biophysics, University of Iowa Carver College of Medicine, Iowa City, IA, 52242, USA; 3 Interdepartmental PhD Program in Genetics, University of Iowa Carver College of Medicine, Iowa City, IA, 52242, USA

**Keywords:** Targeted genomic enrichment, Sequence capture, Massively parallel sequencing, Illumina

## Abstract

**Background:**

Solution-based targeted genomic enrichment (TGE) protocols permit selective sequencing of genomic regions of interest on a massively parallel scale. These protocols could be improved by: 1) modifying or eliminating time consuming steps; 2) increasing yield to reduce input DNA and excessive PCR cycling; and 3) enhancing reproducible.

**Results:**

We developed a solution-based TGE method for downstream Illumina sequencing in a non-automated workflow, adding standard Illumina barcode indexes during the post-hybridization amplification to allow for sample pooling prior to sequencing. The method utilizes Agilent SureSelect baits, primers and hybridization reagents for the capture, off-the-shelf reagents for the library preparation steps, and adaptor oligonucleotides for Illumina paired-end sequencing purchased directly from an oligonucleotide manufacturing company.

**Conclusions:**

This solution-based TGE method for Illumina sequencing is optimized for small- or medium-sized laboratories and addresses the weaknesses of standard protocols by reducing the amount of input DNA required, increasing capture yield, optimizing efficiency, and improving reproducibility.

## Background

In the post-genome era great strides have been made in DNA sequencing technologies. Massively parallel sequencing (MPS) has facilitated the discovery of novel disease genes [1,2] as well as improved diagnostics for inherited disorders such as non-syndromic hearing loss [3] and cancer [4].

While it is clear that in the near future whole genome sequencing (WGS) will become routine, it is currently not feasible for two primary reasons: (1) the cost of sequencing the entire genome with an accuracy level sufficient to call all variants is still prohibitively expensive; and (2) the interpretation of the significance of these variants remains extremely challenging. Targeted genomic enrichment (TGE) approaches were developed to address these issues by reducing cost and variant analysis complexity by screening specific disease-associated genomic regions. Essentially, TGE is a whole genome amplification followed by isolation of specific regions of the genome. Whole exome sequencing (WES) is simply a TGE of all exons in the genome.

TGE may be performed in solution (solution-based targeted genomic enrichment, solution-based TGE, [5]), or on an array platform (solid-phase targeted genomic enrichment, solid-phase TGE, [6]). Although solid-phase TGE was developed first, unlike solution-based TGE it requires expensive hybridization equipment and is less scalable. While some studies have shown that solid-phase TGE provides more even coverage and a greater sensitivity for single nucleotide variants [7], solution-based TGE with ultra-long (>100 bp) oligonucleotides as used in Agilent’s SureSelect system provides a greater sensitivity for indels [8]. However, what may be the primary consideration for a small-to-medium sized laboratory wishing to perform TGE may be the expense associated with ancillary equipment. In the case of solution-based TGE the most expensive ancillary equipment required is a PCR machine. The combination of scalability and little-to-no infrastructure investment has made solution-based TGE the preferred method for TGE in many laboratories.

Current solution-based TGE protocols would benefit from improvement in several areas. First, they should be made more efficient by modifying or eliminating time consuming steps. Second, yield should be maximized to reduce input DNA and excessive PCR cycling. Third, they must be made reliably reproducible. Several improved protocols have been reported for solution-based TGE. In the first protocol modification, several library preparation steps were optimized including efficiency of adaptor ligation [9], but this protocol did not improve overall yield significantly enough to reduce the amount of input DNA. In the second protocol modification, a methodology was developed for high-throughput solution-based TGE using automation equipment at a large genome center [10], however this protocol is not amenable to implementation in small- or medium-sized laboratories. Another recent report increased multiplexing ability but still required 2 micrograms of input DNA and prepared libraries for SOLiD sequencing [11]. Here we report an optimized protocol for fast, reproducible and inexpensive targeted genomic capture for the small- or medium-sized laboratory.

## Results

We developed a solution-based TGE method for downstream Illumina sequencing that incorporates improvements proposed by Fisher et al., 2011 and Mamanova et al., 2010, in a non-automated workflow. In all cases we add standard Illumina barcode indexes during the post-hybridization amplification to allow for sample pooling prior to sequencing. We have validated this method on > 150 libraries using custom target designs ranging from 200 Kb to 1.1 Mb and the whole exome (50 Mb). Here we present results from 44 consecutive libraries prepared with this method and targeting the same genomic region comprising 350,160 bp. This method utilizes Agilent SureSelect baits, primers and hybridization reagents for the capture, off-the-shelf reagents for the library preparation steps, and adaptor oligonucleotides for Illumina paired-end sequencing purchased directly from an oligonucleotide manufacturing company.

### Library preparation and yield

As described previously [10], immediately after shearing genomic DNA, SPRI beads are added to the tube containing the DNA sample and maintained throughout the successive library preparation steps including end-repair, A-tailing, and adaptor ligation. After each incubation, a purification is performed in the same tube. In order to allow the DNA to re-associate with the SPRI beads, a highly charged NaCl-PEG buffer is introduced. The ratio of this buffer to the DNA solution determines the size of DNA that will associate with the beads. We have found that this method of purification greatly reduces DNA loss (~8.6% loss after each purification step) as compared to column purification (~20.5% loss after each purification step) and standard SPRI purification (~18.8% loss after each purification step), as detailed in Additional file 1.

We found that after shearing with the Covaris, a tight size range is attained, and this size range can be maintained with a 1:1 ratio of NaCl-PEG buffer to sample, negating the need for any size selection with gel electrophoresis, bead-based selection, or specialized equipment. The primary benefit of this method is that DNA loss is minimized during elution or tube transfers, as the sample is maintained in the same tube. Additionally, the chances for sample mix-up are minimized as there are fewer tube transfers in the protocol (only 3 versus the standard 9 prior to the hybridization step). Immediately prior to the pre-hybridization amplification, DNA is eluted off the beads. Standard SPRI purifications are performed after both pre- and post- hybridization amplifications and after sequence capture. In all cases when the DNA sample is isolated from the SPRI beads, we perform a double elution to increase yields. All column-based purifications have been removed, as they are associated with an increased loss of DNA and present chances for sample-mix ups. These improvements have allowed us to reduce sample input.

### Starting input and yield

Standard protocols require 3,000 ng of genomic DNA (gDNA) per sample. During the development of this protocol we noted that our yields were consistently high after the pre-hybridization PCR. In order to proceed with hybridization and subsequent sequence capture, 500 ng of DNA is required. Using 3,000 ng of input gDNA and the standard Agilent SureSelect protocol (v1.0 September 2009) yielded on average 650 ng of adaptor-ligated DNA when performing up to 18 amplification cycles during the prehybridization PCR. Current Agilent protocols recommend 4–6 cycles (v1.2 May 2011), however in our experience up to 12 cycles are required to obtain enough DNA for hybridization. Using the protocol described here, we have been able to reduce the number of amplification cycles required to 6 cycles while increasing the yield of adaptor-ligated DNA to an average of 2,432 ng (Table 1).

**Table 1:** Sequencing results for the same sample sequenced with either 3,000 ng or 500 ng input DNA

**Metric**	**Sample 1A – 3,000 ng**	**Sample 1D – 500 ng**	**Average (Std Dev)**
DNA after prehyb PCR (ng)	2,734	2,625	2,432 (541)
Total sequencing reads	39,964,814	28,476,648	37,685,938 (27,502,720)
% of reads mapped	97.70%	98.40%	94.5% (4.9%)
% duplicate reads	56.80%	44.80%	53.7% (16.3%)
% of reads overlapping target	62.90%	54.50%	61.7% (3.7%)
% of targeted bases covered at 10X	98.10%	98.50%	97.8% (0.5%)
Avg depth of coverage of target regions	4,692	3,660	5,826 (4,200)

Average includes data from all samples including 1A and 1D (n = 44). Targeted region size was 350,160 bp. Distribution of coverage was also similar ( Additional file 1).

**Figure 1:**
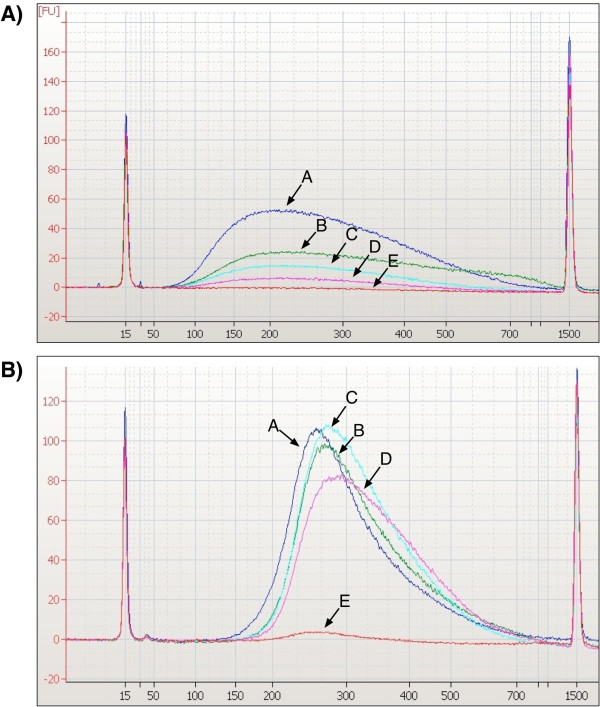
**Reduced requirement for input DNA using this method.** A single gDNA sample was taken through the protocol using either 3,000 ng (curve A), 1,500 ng (curve B), 750 ng (curve C), 500 ng (curve D), and 375 ng (curve E) starting material and 6 cycles of PCR amplification. **A**) Bioanalyzer trace after shearing step, **B**) Bioanalyzer trace after pre-hybridization amplification showing no detriment to overall library preparation yield for starting material greater than 500 ng.

We also tested lower amounts of starting input DNA, including 1,500, 1,000, 750, 500 ng or 375 ng, using the same number of PCR cycles in all cases (6 cycles) and could achieve yields comparable to 3,000 ng input with starting amounts as low as 500 ng (Figure 1). The yield using 375 ng of starting DNA was reduced (Figure 1), but the number of amplifications can be increased during the prehybridization PCR to obtain adequate yields (data not shown), although PCR cycles should always be minimized to reduce the number of PCR duplicates. By comparing the final sequencing enrichment statistics from the same sample prepared with either 3,000 ng or 500 ng starting material, the reduced input does not have any significant effect on target enrichment or library complexity (Table 1). Similar results were achieved when using gDNA from blood (extracted using a PureGene Kit) or saliva (extracted with a Genotek Kit) (data not shown).

### Handling and efficiency

The “with-bead” method of purification was faster in our hands than column purification or standard SPRI purification. Based on these improvements, average hands-on time for a set of 10 captures breaks down to 11 hours over three days, as follows: (1) Covaris shearing to hybridization-ready sample (includes lyophilization), 6 hours day 1; (2) hybridization set up, 1 hour day 2; (3) sequence capture, post-hybridization amplification, 4 hours, day 3.

The use of off-the-shelf enzymes has two primary advantages: (1) because the reagents are not kitted, it allows for excess reagent availability in the case of a preparation failure, and (2) it allows for modification of the protocol.

### Reproducibility

**Figure 2:**
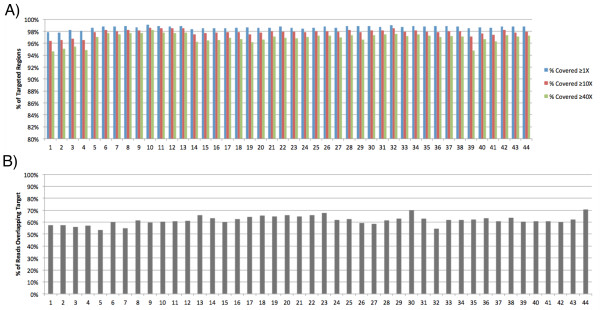
**Sequencing results show high reproducibility of the method for 44 consecutive samples prepared using this protocol.** Both **A**) high capture efficiency, and **B**) high coverage of targeted regions are maintained and are reproducible. Data shown are for enrichment of 350,160 bp.

Figure 2 shows the high reproducibility of enrichment quality when a relatively small region of interest (350,160 bp) is targeted. On-target capture efficiency is primarily a result of the library preparation process and this method has a high reproducibility. In addition, a consistently high proportion of targeted regions were covered at 1X, 10X, and 40X (Figure 2).

## Discussion

Since TGE was first described, advances have been made in library preparation methods for TGE, but none of the advances have focused on the workflows necessary for small- to medium-sized laboratories nor have they been adapted to the most widely used sequencing platform, Illumina. Nijman et al. present a method where up to 96 samples can be multiplexed, but the protocol is specific to SOLiD sequencing and solid-phase TGE [12]. Another recent publication allows pre-capture multiplexing of up to 12 samples but the adaptors described can only be used with single-end Illumina sequencing [13]. A more recent protocol, although providing the ability to perform precapture multiplexing on both solid-phase and solution-based TGE is optimized for SOLiD sequencing [11]. None of these protocols incorporates methods to decrease input by maximizing yield or reducing hands-on time necessary for a small- or medium-sized laboratory. Natsoulis et al. describe a unique approach whereby complementary DNA oligos are manufactured using a freely available resource and precapture multiplexing is used [14], however the majority of investigators in medium-sized laboratories do not have bioinformatics ability to evaluate robustness of TGE designs, a component which is included in commercial TGE design options.

In designing this protocol, we used key features in two previously published optimized protocols, as well as the manufacturer’s protocol to develop our own protocol for solution-based TGE, which is suitable for library preparation for paired-end Illumina sequencing and enrichment for small targeted regions or the whole exome. Most protocols for solution-based TGE require 3,000 ng of starting input DNA. Human genomic DNAs are often in short supply and therefore must be used sparingly. A primary goal of this new protocol was to reduce gDNA input and therefore save precious samples. By making several changes we were able to reduce the amount of DNA required to 500 ng with no detrimental effects on library preparation yield or downstream sequence output (Figure 1 and Table 1). This protocol has the added benefit of reducing the number of cycles required during PCR steps, as excessive amplification can reduce library complexity due to decreased representation of genomic regions with high GC content. This protocol is scalable for a medium-sized lab to easily perform 10 library preparations in three days with a single technician.

We use off-the-shelf enzymes for all of the pre-hybridization steps and amplification steps primarily because kitted enzymes are proprietary, do not list exact enzymatic properties (i.e. units per uL) and are therefore difficult to adjust and troubleshoot experimentally. Importantly, when ordering SureSelect baits, the customer has the option to request only the baits and hybridization reagents as opposed to the full kit including all enzymes (“XT”). Therefore for use with this protocol we recommend the non-XT kits to maximize savings. We use the primers provided in the Agilent kits, but we order the Illumina adaptors oligonucleotides and primers directly from an oligonucleotide provider as we use excess amounts of these reagents. It is possible to adapt this protocol, or steps from this protocol, to increase yield for SOLiD library preparation (data not shown). In addition, we feel that this protocol could easily be adapted to custom-designed DNA baits and precapture multiplexing, if required.

## Conclusion

This method provides small-to-medium sized laboratories a viable option for custom or whole exome enrichment of precious DNA samples and addresses the primary weaknesses associated with standard protocols.

## Methods

Oligonucleotides were ordered from IDT Inc. – oligonucleotide sequences available upon request from Illumina, Inc. Enzymes are from New England Biosciences. All sequencing was performed on the Illumina HiSeq using a variable number of multiplexed samples per lane. See Additional file 2 for complete protocol.

## Competing interests

The authors declare that they have no competing interests.

## Authors’ contributions

All authors designed the study, performed analyses and drafted the manuscript. AES and MSH performed sequence capture and designed the protocol. RJHS conceived of the study. All authors have read and approved the final manuscript.

## Supplementary Material

Additional file 1: Evaluation of purification methods and mean coverage comparison. Click here for file

Additional file 2: MORL solution-based targeted genomic enrichment protocol – Illumina sequencing with multiplexing. Click here for file
