# Supplementary material for: Solution-based targeted genomic enrichment for precious DNA samples
Source: BMC Biotechnol. 2012 May 4;12:20. doi: 10.1186/1472-6750-12-20 (PMC3561655; doi:10.1186/1472-6750-12-20)
Supplement: Additional file 1: — Evaluation of purification methods and mean coverage comparison. [file 1472-6750-12-20-S1.pdf]

MORL Solution-Based Targeted Genomic Enrichment Protocol – Illumina Sequencing with Multiplexing

NOTES

- Eppendorf LoBind Tubes should be used for all steps to ensure minimal sample retention
- Use ABI Veriti thermal cycler and tube-strips with dome caps for hybridization (GeneMate p/n 3-3014-1)
- In all cases, use molecular biology grade water (distilled DNAse-RNAse free water, not DEPC treated)
- New England Biosciences enzymes are used
- Agilent oligonucleotides are provided in SureSelect kit. Illumina oligonucleotides including universal adaptors and barcoding primers are ordered from IDT. Oligonucleotide sequences available upon request from Illumina, Inc.
- Recipe for PEG-SPRI buffer is at end of protocol.

PART 1: LIGATION OF ADAPTORS AND AMPLIFICATION

I. Ensure quality of DNA samples to be used

To ensure quality library preparation, high quality genomic DNA should be used. This DNA can be prepared via a variety of methods, but DNA from whole blood and extracted with the PureGene kit has performed well in our lab. We have also used DNA extracted from saliva.

1. Check quality of DNA on a gel to ensure that DNA is not previously fragmented as this will affect shearing size. Using a 1% agarose gel, run 1 uL of >50 ng/uL DNA sample with 2uL dye and 7 uL of water at 100 volts for 1 hour. Ensure that band of gDNA is greater than the highest ladder marker (i.e. greater than 5kb).
2. Using a spectrophotometer or nanodrop, ensure that DNA sample 260/280 ratio is between 1.8 and 2.0. DO NOT use spectrophotometer for DNA quantification as this will over-estimate DNA concentration by up to 50%
3. Quantify DNA using Qubit or PicoGreen system, ensuring that %CV is <10%.

II. Shearing samples to 100-300bp

The Covaris sonication system is used to ensure a tight size distribution of sheared DNA. This, along with SPRI bead purifications, negates the need for a size-selection step.

Dilute between **375ng to 3,000 ng** genomic DNA to a total volume of **120 µl** with LoTE. Use the same amount of DNA for each sample to be prepped together to ensure that the same conditions are appropriate for all samples.

4. Mix and transfer to a 6mmx16mm AFA Covaris tube.
5. Shear with a Covaris, using the settings for **2 cycles of 60 seconds**:

Duty cycle	**20%**
Intensity	**5**
Cycle/burst	**200**

6. After shearing, remove vial from the Covaris. Carefully remove vial top and transfer sample into a fresh **1.5 ml LoBind eppendorf tube**. Keep samples on ice whenever possible.
7. OPTIONAL: Run 1 µl on an **Agilent Bioanalyzer DNA 1000** chip to ensure proper shearing. If preparing 6 samples or less, a 1.5µl aliquot of each sample can be taken and retained until the Bioanalyzer step after the pre-hybridization PCR and all samples can be run on the same chip (each chip can run 12 samples).

III. Addition of SPRI Beads and SPRI Purification after Shearing

A 1.8X volume of SPRI beads is added at this step, and these beads will be maintained in the reaction mix until the pre-hybridization PCR. Allow SPRI beads to come to room temperature for at least ***30 minutes before use****. Vortex SPRI beads well prior to use so they appear homogeneous.*

1. Add **216 µl** of SPRI beads (1.8X volume) to the **120 µl** of sheared DNA.
2. Vortex SPRI-DNA solution and leave at room temperature for **5 minutes**.
3. Place tubes in a magnetic rack and leave for up to **5 minutes** or until sample is clear.
4. Carefully remove the clear supernatant from the tubes and discard.
5. Dispense **700 µl of 70 % ethanol** into each tube while in the magnetic rack taking care not to disturb the magnetic beads. Aspirate and discard ethanol.
6. Repeat the ethanol wash once again (total of two washes).
7. Dry the samples on a heat block (keep the lid of the tube open) at 37 °C for 5 to 10 minutes or until the residual ethanol has evaporated. Do not over-dry samples (excessive cracking of beads is not desirable) as this will reduce elution

efficiency

8. Add **45 µl** of molecular biology grade water, vortex and incubate at room temperature for **2 minutes** before proceeding with the next step.

IV. End Repair

During this step, the overhanging DNA strands are removed to create double-stranded fragments with blunt ends using the 3' to 5' exonuclease activity of Klenow and the 5' polymerase activity of DNA polymerase.

1. Prepare a master mix containing the following reaction mix plus a 10 % excess

	Lot	1X	___X	+10%	Added?
Water		**30 µl**			
10x T4 DNA ligase buffer with 10mM ATP		**10 µl**			
10mM dNTP mix		**4 µl**			
3U/ µl T4 DNA polymerase		**5 µl**			
5U/ µl Klenow DNA polymerase		**1 µl**			
10U/ µl T4 PNK		**5 µl**			

2. Vortex and aliquot **55 µl** of master mix into each sample tube containing the beads and **45 µl of eluate** from the previous step. Mix well and spin down.
3. Incubate for **30 minutes** at RT (**25°**).

V. SPRI Purification after End Repair

1. Add **100 µl PEG 8000/NaCl (PEG-SPRI binding buffer)** solution to the tube containing **100uL of product (1:1 ratio)** and SPRI beads.
2. Vortex and leave at room temperature for **5 minutes**.
3. Place tubes in a magnetic rack. Leave for **5 minutes** or until sample is clear.
4. Carefully remove the clear supernatant from the tubes and discard.
5. Dispense **700 µl of 70 % ethanol** into each tube while in the magnetic rack taking care not to disturb the magnetic beads. Aspirate and discard ethanol.
6. Repeat the ethanol wash once again (total of two washes).
7. Dry the samples on a heat block (keep the lid of the tube open) at **37 °C for 5 to 10 minutes** or until the residual ethanol has evaporated.
8. Add **32 µl** of molecular biology grade water, vortex and incubate at room temperature for **2 minutes** before proceeding with the next step.

VI. 'A' Base Addition to the 3' End of dsDNA fragments

During this step, the Klenow fragment is used to add a single 'A' base to the 3' ends of the blunt dsDNA fragments. This allows these fragments to ligate to the universal adaptors which have a single 'T' base overhang on their 3' end.

1. Prepare a master mix containing the following reaction mix, plus a 10 % excess:

	Lot	1X	____X	+10%	Added?
10x Klenow buffer		**5 µl**			
1 mM dATP		**10 µl**			
5U/uL Klenow Exo (3' 5o 5' exo minus)		**3 uL**			

2. Vortex and aliquot **18 µl** of master mix into each sample tube containing the beads and **32 µl of end- repaired** sample.
3. Mix well and spin down. Volume is **50uL total**.
4. Incubate for **30 minutes at 37 °C** in a hot block.

VII. SPRI Purification after 'A' base addition

1. Add **50 µl PEG-SPRI binding buffer** to the tube containing **50uL of product (1:1 ratio)** and SPRI beads.
2. Vortex and leave at room temperature for **5 minutes**.
3. Place tubes in a magnetic rack. Leave for **5 minutes** or until sample is clear.
4. Carefully remove the clear supernatent from the tubes and discard.
5. Dispense **700 µl of 70 % ethanol** into each tube while in the magnetic rack taking care not to disturb the magnetic

beads. Aspirate and discard ethanol.
6. Repeat the ethanol wash once again (total of two washes).
7. Dry the samples on a heat block (keep the lid of the tube open) at **37 °C for 5 to 10 minutes** or until the residual ethanol has evaporated.
8. Add **10 µl** of molecular biology grade water, vortex and incubate at room temperature for **2 minutes** before proceeding with the next step.

VIII. Ligation of Adapters to DNA Fragments

1. Prepare a master mix containing the following reaction mix, plus a 10 % excess:

	Lot	1X	____X	+10%	Added?
2 x DNA ligase buffer		**21 µl**			
Illumina PE Adapter oligo mix		**9 µl**			
2,000 U/ µl T4 DNA ligase		**10 µl**			

2. Mix and aliquot **40 µl master mix** into each sample tube containing the **10 µl of A-tailed sample**. Mix and spin down. Volume is **50uL total**.
4. Incubate for **15 minutes** at RT (**25°)**.

IX. SPRI Purification and DNA elution from SPRI beads

At this point, the DNA will be eluted off of the beads and the beads will be removed prior to the PCR. A double elution is performed at this step to ensure that all DNA is eluted from the beads.

1. Add **50 µl PEG-SPRI binding buffer** to the tube containing **50uL of product (1:1)** and SPRI beads.
2. Vortex and leave at room temperature for **5 minutes**.
3. Place tubes in a magnetic rack. Leave for **5 minutes** or until sample is clear.
4. Carefully remove the clear supernatent from the tubes and discard.
5. Dispense **700 µl of 70 % ethanol** into each tube while in the magnetic rack taking care not to disturb the magnetic beads. Aspirate and discard ethanol.
6. Repeat the ethanol wash once again (total of two washes).
7. Dry the samples on a heat block (keep the lid of the tube open) at **37 °C for 5 to 10 minutes** or until the residual ethanol has evaporated.
8. Add **25 µl** of molecular biology grade water, vortex and incubate at room temperature for **2 minutes**.
9. Place tubes into the magnetic rack and leave for 2-3 minutes or until sample is clear. Carefully remove the water and retain in a new 1.5 ml lo-bind Eppendorf tube.
10. Repeat step 8-10 once more, retaining the water in the same 1.5 ml tube. Total volume of eluate should be **50 µl**.

X. Pre-hybridization PCR

A minimum number of PCR cycles should be used to prevent PCR-induced bias and reduced library complexity. If a smaller starting amount of DNA is used, 8 cycles may be necessary. In almost all cases 6, or even 4 PCR cycles are sufficient. 500 ng DNA is required for hybridization, and so a goal is to aim for 1,000 ng DNA after this step as quantified on the Bioanalyzer. To increase efficiency, amplify each 50 µl adapter- ligated library by dividing between 4 PCR reactions.

1. Prepare a master mix containing the following reaction mix per sample, plus a 10 % excess:

	Lot	1X	____X	+10%	Added?
Illumina InPE1.0 (F) primer		**4 µl**			
SureSelect PreCapture PCR (R) primer		**4 µl**			
2X Phusion HF master Mix		**100 µl**			
Water		**42 µl**			

2. Mix and aliquot **150 µl** of master mix into each **50 µl adapter-ligated library**. Mix and spin down.
3. Aliquot equally into **four 200 µl PCR tubes** (**50 µl each**), and perform the following temperature cycling for **6 cycles**:

**Temp**	**Time**	**Cycles**
98 °C	2 min	1
98 °C	20 sec	6
65 °C	30 sec	
72 °C	30 sec	

72 °C	5 min	1
4 °C	hold	1

Machine Used ____________________ Protocol ________________

XI. Post-Amplification SPRI bead cleanup

*Allow SPRI beads to come to room temperature for at least **30 minutes**. Vortex SPRI beads well prior to use so they appear homogeneous.*

1. Spin PCR tubes down and combine 4 tubes per sample into a single 1.5mL Eppendorf Lo-bind tube (200 uL total of sample)
2. Add **360 µl** of SPRI beads to the **200 µl** of PCR sample.
3. Vortex and leave at room temperature for **5 minutes**.
4. Place tubes in a magnetic rack. Leave for **5 minutes** or until sample is clear.
5. Carefully remove the clear solution from the tubes and discard.
6. Dispense **700 µl of 70 % ethanol** into each tube while in the magnetic rack taking care not to disturb the magnetic beads. Aspirate and discard ethanol.
7. Repeat the ethanol wash once again (total of two washes).
8. Dry the samples on a heat block (keep the lid of the tube open) at **37 °C for 5 to 10 minutes** or until the residual ethanol has evaporated.
9. Add **25 µl** of molecular biology grade water, vortex and incubate at room temperature for **2 minutes**.
10. Place tubes into the magnetic rack and leave for 2-3 minutes or until sample is clear. Carefully remove the water and retain in a new 1.5 ml lo-bind Eppendorf tube.
11. Repeat step 8-10 once more, retaining the water in the same 1.5 ml tube. Total volume of eluate should be **50 µl**.

--> *Quantify **1 µl** of library using an **Agilent DNA 1000 chip** on a Bioanalyzer 2100 and proceed to hybridization*

PART 2: HYBRIDIZATION AND SEQUENCE CAPTURE

During this part of the protocol, the 24 hour hybridization will be performed by combining the RNA baits with the prepped adaptor-ligated DNA library. This will be followed by a streptavidin-bead "capture" and then by an amplification.

I. Concentration of DNA

500 ng of DNA is required to proceed at this step and 3.4 uL is needed. Therefore, DNA must be at a concentration of ≥147ng/uL and in almost all cases, DNA needs to be lyophilized.

1. Cut lids off of 1.5mL Eppendorf tubes. Cover opening with parafilm and poke a hole in the middle.
2. Place in vacuum concentrator, and balance tubes.
3. Run with heat and vacuum on until completely lyophilized – takes approximately 1-2 hours. Make sure to not over-dry samples. Check often.
4. Reconstitute to 147 ng/uL concentration with nuclease-free water based on concentration from previous bioanalyzer run.

II. Hybridize the Library

Four components will be prepared: the HYB BUFFER, BLOCK MIX, CAPTURE BAITS and DNA LIBRARY. All components will be heated to 65°C in a PCR machine prior to combining into a single PCR well. It is best to perform the hybridization steps with a partner, as it is imperative to move quickly when the PCR machine is running to prevent excess evaporation.

1. Prepare Hybridization Buffer (**HYB BUFFER): Mix at Room Temperature**

	Lot	1X (includes excess)	____X	Added?
SureSelect Hyb #1		**25 µl**		
SureSelect Hyb #2 (red cap)		**1 µl**		
SureSelect Hyb #3 (yellow cap)		**10 µl**		
SureSelect Hyb #4		**13 µl**		

If precipitate forms, warm the hyb buffer at 65 degrees for 5 minutes

2. Prepare the Block Mix **(BLOCK MIX): Mix at Room Temperature**

	Lot	1X	____X	+10%	Added?
SureSelect Indexing Block #1 (green cap)		**2.5 µl**			

SureSelect Block #2 (blue cap)		**2.5 µl**			
SureSelect Indexing Block #3 (brown cap)		**0.6 µl**			

3. Prepare the Capture Baits (**CAPTURE BAITS**): **Keep Tubes on Ice Until Step 2.II.8**

   a. Prepare RNase Block as per table: <3MB libraries (1:9 dilution) **note that there is a different dilution to be used for libraries >3MB (i.e. exomes)*

	Lot	1X	____X	Added?
RNase Block		**1uL**		
Nuclease-Free Water		**9uL**		

   b. Prepare SureSelect RNA Capture library (BAITS) as per table (total for each sample is 7uL): **note that there is a different dilution to be used for libraries >3MB (i.e. exomes)*

Lot (ELID #)	Vol of SureSelect Library (Baits) 1X	____X	Added?	Vol of Diluted RNase Block	____X	Added?
	**2uL**			**5uL**		

4. Prepare the Prepped DNA Library (**LIBRARY**): **Prepare on Ice**
   a. Your library should be 147 ng/uL (see step 2.II)
   b. Add 3.4 uL of the 147 ng/uL prepped library to **Row B**. Put each sample in a separate well
   c. Add 5.6 uL of the BLOCK mix (2.II.2) to each well in **Row B**. Mix well by pipetting up and down and change tips between each well.
   d. Seal tubes with caps.
5. Set up PCR Tubes for hybridization
   a. Place PCR rack on ice
   b. Prepare three rows of PCR tubes with Snap-off lids, labeled Row A, B, C. Ensure there are enough for each sample.
   c. See Figure (PAGE 5) as a reference for the next steps.
6. Place the 3 rows of tube strips into PCR machine and run the following program using a heated lid:

**Temp**	**Time**	**Cycles**
95 °C	5 min	1
65°C	Hold – 24 hours	1

Machine Used ____________________ Protocol ________________

7. Add HYB BUFFER:
   a. After machine reaches 65°C, proceed with the next step.
   b. With PCR machine still running, open lid and remove lids from Row A.
   c. Add **40 uL of HYB BUFFER** to **Row A**. Maintain the machine at 65°C.
   d. Reseal lids.
   e. Close PCR machine and incubate for 5 minutes before proceeding to next step.
8. Add RNA BAITS:
   a. With PCR machine still running, open lid and remove lids from Row C.
   b. Add **7 uL of RNA BAITS** to **Row C**. Maintain the machine at 65°C.
   c. Reseal lids.
   d. Close PCR machine and incubate for 2 minutes before proceeding to next step.
9. TRANSFER STEP: Perform these steps while maintaining the machine at 65°C and working as quickly as possible to prevent excess evaporation.
   a. Take **13 uL of HYB BUFFER from Row A and add it to Row C** (13 uL A --> C)
   b. Take **9 uL (entire volume) of LIBRARY from Row B and add it to Row C** (9 uL B --> C)
   c. Pipette up and down 8-10 for each sample. Change pipettes between sample.
   d. Row C (HYB MIXTURE) should now contain ~29 uL
   e. Close PCR machine and incubate for 24 hours at 65°C.

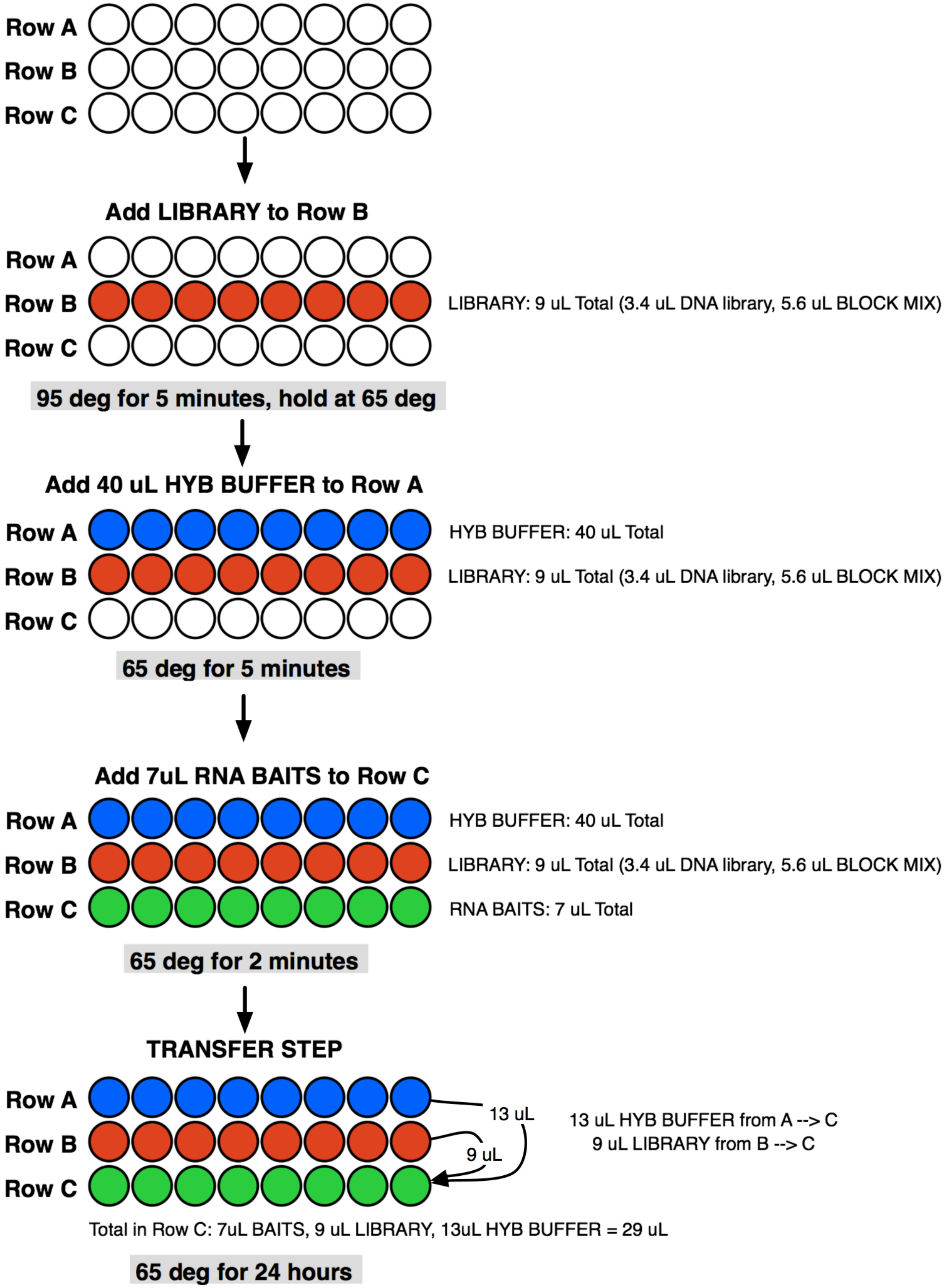
Row A
Row B
Row C
Add LIBRARY to Row B
Row A
Row B
Row C
LIBRARY: 9 uL Total (3.4 uL DNA library, 5.6 uL BLOCK MIX)
95 deg for 5 minutes, hold at 65 deg
Add 40 uL HYB BUFFER to Row A
Row A
Row B
Row C
HYB BUFFER: 40 uL Total
LIBRARY: 9 uL Total (3.4 uL DNA library, 5.6 uL BLOCK MIX)
65 deg for 5 minutes
Add 7uL RNA BAITS to Row C
Row A
Row B
Row C
HYB BUFFER: 40 uL Total
LIBRARY: 9 uL Total (3.4 uL DNA library, 5.6 uL BLOCK MIX)
RNA BAITS: 7 uL Total
65 deg for 2 minutes
TRANSFER STEP
Row A
Row B
Row C
13 uL
9 uL
13 uL HYB BUFFER from A --> C
9 uL LIBRARY from B --> C
Total in Row C: 7uL BAITS, 9 uL LIBRARY, 13uL HYB BUFFER = 29 uL
65 deg for 24 hours

III. Bead Capture

This step uses streptavidin-coated beads (DynaBeads) to selectively capture the biotinylated RNA-bait-DNA complex. The beads need to be washed prior to use.

1. Prewarm SureSelect Wash Buffer #2 at **65°C** in a water bath for use in Step 2.III.3.h
2. Prepare the DynaBeads:
    a. Vigorously resuspend the DynaBead Streptavidin (Invitrogen) magnetic beads on a vortex mixer.
    b. For each hybridization, add **50 uL Dynal magnetic beads** to a 1.5 mL Lo-bind tube.
    c. Wash the beads (note that you can wash beads for up 5 samples in a single 1.5 mL tube, i.e. 250 uL beads washed with 1,000 uL Binding buffer, then divide into separate tubes after resuspension with 200uL per tube):
        i. Add **200 uL SureSelect Binding Buffer** to each tube.
        ii. Mix the beads on a vortex mixer for 5 seconds.
        iii. Put the tubes in the magnetic separator and wait for solution to clear (1-2 min).
        iv. Remove and discard the supernatant.
        v. Repeat steps i-iv for a total of 3 washes.
    d. Resuspend the beads in **200 uL SureSelect Binding buffer**.
3. Hybrid Capture with SureSelect
    a. Add sample directly from PCR machine to the DynaBead solution. Estimate volume retained after hybridization. There should be >20 uL in all cases. If excessive evaporation has occurred, repeat hybridization.
    b. Invert 5 times to mix.
    c. Incubate the hybrid-capture/bead solution on a Nutator for **30 minutes** at **room temperature**. Ensure proper mixing is occurring.
    d. Briefly spin in a centrifuge. Separate the beads and buffer on the magnetic separator and remove the supernatant.
    e. Resuspend the beads in **500 uL SureSelect Wash Buffer #1** by mixing on a vortex mixer for 5 seconds.
    f. Briefly spin in a centrifuge. Separate the beads and buffer on a magnetic separator and remove the supernatant.
    g. Wash the beads:
        i. Resuspend the beads in **500 uL of prewarmed SureSelect Wash Buffer #2.**
        ii. Mix on a vortex mixer for 5 seconds to resuspend the beads.
        iii. Incubate the samples for **10 minutes** at **65°C.** Occasionally mix on a vortex mixer.
        iv. Briefly spin in a centrifuge. Separate the beads and buffer on a magnetic separator and remove the supernatant.
        v. Repeat steps i-iv for a total of 3 washes
        vi. Make sure all of the wash buffer has been removed
    h. Add **50 uL of SureSelect Elution Buffer** and mix on a vortex mixer for 5 sec to resuspend the beads.
    i. Incubate the samples for **10 minutes** at **room temperature**. Occasionally mix on a vortex mixer.
    j. Separate beads and buffer on a magnetic separator. The supernatant now contains the captured DNA.
    k. Use a pipette to transfer the supernatant to a new 1.5 mL Lo-bind tube. Add **50 uL SureSelect Neutralization Buffer** to the capture DNA. Briefly mix on a vortex mixer.

IV. Post-Capture SPRI bead cleanup

*Allow SPRI beads to come to room temperature for at least **30 minutes**. Vortex SPRI beads well prior to use so they appear homogeneous.*

4. Add **180 µl** of SPRI beads to the **100 µl** of DNA sample in a 1.5 ml Lo-bind Eppendorf tube.
5. Vortex and leave at room temperature for **5 minutes**.
6. Place tubes in a magnetic rack. Leave for **5 minutes** or until sample is clear.
7. Carefully remove the clear solution from the tubes and discard.
8. Dispense **500 µl of 70 % ethanol** into each tube while in the magnetic rack taking care not to disturb the magnetic beads. Aspirate and discard ethanol.
9. Repeat the ethanol wash once again (total of two washes).
10. Dry the samples on a heat block (keep the lid of the tube open) at **37 °C for 5 to 10 minutes** or until the residual ethanol has evaporated.
11. Add **15 µl** of molecular biology grade water, vortex and incubate at room temperature for **2 minutes**.
12. Place tubes into the magnetic rack and leave for 2-3 minutes or until sample is clear. Carefully remove the water and retain in a new 1.5 ml lo-bind Eppendorf tube.
13. Repeat step 8-10 once more, retaining the water in the same 1.5 ml lo-bind tube. Total volume of eluate should be **30 µl**.

V. Post-hybridization PCR and addition of indexing Barcodes

Determine before-hand which indexes to use based on the number of samples to be pooled. When experiments require the use of fewer than 12 samples in a lane, select indexes to ensure optimum cluster discrimination during sequencing by having a different base at each cycle of the index read. For example, for 2 samples: Index #6 and Index #12. For 6 samples, Indexes #2, #4, #5, #6, #7, #12. Only use Herculase Polymerase and dNTPs during this step.

1. Prepare a master mix containing the following reaction mix per sample, plus a 10 % excess:

	Lot	1X	____X	+10%	Added?
SureSelect Indexing Post-Capture (F) Primer		**1 µl**			
Herculase dNTP mix (100 mM, 25mM of each)		**0.5 µl**			
5X Herculase II Reaction Buffer		**10 µl**			
Herculaes II Fusion DNA Polymerase		**1 µl**			
Nuclease-Free Water		**22.5 µl**			

2. Add **35 µl** of master mix to each PCR tube.
3. Add **1 µl** of each index SureSelect Indexing Primer (R) from the Illumina Multiplexing Sample Kit to each tube.

Indexes used:

**SAMPLE**	**INDEX #**

**SAMPLE**	**INDEX #**

4. Add **14 µl** of each DNA sample to each tube. Mix well by pipetting.
5. Put the tubes in a thermal cycler and run the following program (# of cycles depends on target size – for <0.5MB, use 16 cycles, for 0.5-1.5MB, use 14):

**Temp**	**Time**	**Cycles**
98 °C	30 sec	1
98 °C	10 sec	14 or 16
57 °C	30 sec	
72 °C	30 sec	
72 °C	5 min	1
4 °C	hold	1

Machine Used ____________________ Protocol ________________

VI. Post-Amplification SPRI Cleanup

*Allow SPRI beads to come to room temperature for at least **30 minutes**. Vortex SPRI beads well prior to use so they appear homogeneous*

1. Add **90 µl** of SPRI beads to the **50 µl** of PCR sample in a 1.5 ml Lo-bind Eppendorf tube.
2. Vortex and leave at room temperature for **5 minutes**.
3. Place tubes in a magnetic rack. Leave for **5 minutes** or until sample is clear.
4. Carefully remove the clear solution from the tubes and discard.
5. Dispense **500 µl of 70 % ethanol** into each tube while in the magnetic rack taking care not to disturb the magnetic beads. Aspirate and discard ethanol.
6. Repeat the ethanol wash once again (total of two washes).
7. Dry the samples on a heat block (keep the lid of the tube open) at **37 °C for 5 to 10 minutes** or until the residual ethanol has evaporated.
8. Add **15 µl** of molecular biology grade water, vortex and incubate at room temperature for **2 minutes**.
9. Place tubes into the magnetic rack and leave for 2-3 minutes or until sample is clear. Carefully remove the water and retain in a new 1.5 ml lo-bind Eppendorf tube.
10. Repeat step 8-10 once more, retaining the water in the same 1.5 ml tube. Total volume of eluate should be 3**0 µl**.

➔ *Quantify **1 µl** of library using an **Agilent High Sensitivity DNA chip** on a Bioanalyzer 2100. Determine concentration by integrating under the curve.*

PEG-SPRI Buffer for with Bead Protocol (20% PEG 8000/2.5M NaCl) for 100 mL solution:

- 20 grams PEG 8000
- 50 mL 5M NaCl solution or 14.6 g solid NaCl
- H20 to bring final volume to 100 mL
- Stir using magnetic bar
- Autoclave using the liquid setting (121degC for 20 min)

*solution may separate during autoclave -- allow to cool then shake back to homogeneous
