# Supplementary material for: Solution-based targeted genomic enrichment for precious DNA samples
Source: BMC Biotechnol. 2012 May 4;12:20. doi: 10.1186/1472-6750-12-20 (PMC3561655; doi:10.1186/1472-6750-12-20)

# SUPPLEMENTARY INFORMATION

# Part 1: EVALUATION OF PURIFICATION METHODS

We evaluated the performance of three methods: standard column purification (Qiagen), standard Solid-Phase Reverse Immobilization (SPRI) bead purification (Agencourt AMPureXP), and “with-bead” SPRI purification as described in this protocol. Our aim was to closely approximated the pre-hybridization library preparation steps by performing 4 purifications repeatedly on the same DNA sample. We performed these purifications in replicates of 3 at each of two different starting DNA amounts – 3,000 ng and 500 ng for a total of 6 samples for each method.

For column purifications we followed the manufacturer’s recommended protocol with an elution of 50 uL. For the standard SPRI bead purifications we again followed the manufacturer’s recommended protocol with an elution of 30 uL. For the “with-bead” SPRI purifications we followed the protocol outlined in the protocol described in this paper with an elution of 30 uL. We took 1.5 uL after each elution and quantified DNA by spectrophotometer (Nanodrop). In the case of the “with-bead” purifications because no elution is performed until the last purification we placed the tube on the magnet to isolate the magnetic beads and removed 1.5 uL directly from the tube before adding the PEG-SPRI buffer to re-associate the beads with the DNA.

Results show that on average with each purification, 20.5%, 18.8%, and 8.6% of DNA is lost with each column, standard SPRI, and “with-bead” SPRI purification, respectively. As shown in the figure below, after four purifications there was 18.2%, 24.6%, and 65.8% DNA remaining after the column, standard SPRI, and “with-bead” SPRI purification, respectively.

#
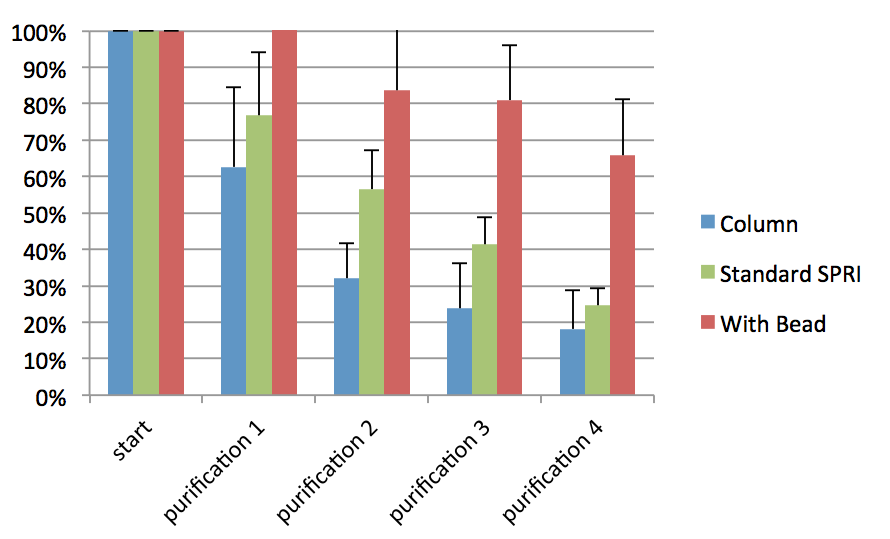


# Part 2: Mean Coverage Comparison

This figure shows an overlapping histogram of mean coverage for all 44 samples (green), and sample 1D (500 ng starting material used). The data shows a very similar deviation from the mean when sample 1D is compared to the average of all others.


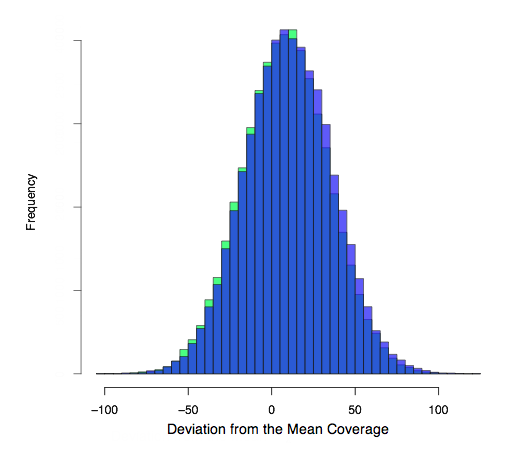

Supplement: Additional file 2: — MORL solution-based targeted genomic enrichment protocol – Illumina sequencing with multiplexing. [file 1472-6750-12-20-S2.doc]
